# General theory of skin reinforcement

**DOI:** 10.1371/journal.pone.0182865

**Published:** 2017-08-10

**Authors:** Ilja L. Kruglikov, Philipp E. Scherer

**Affiliations:** 1 Wellcomet GmbH, Scientific Department, Karlsruhe, Germany; 2 Touchstone Diabetes Center, Department of Internal Medicine, University of Texas Southwestern Medical Center, Dallas, Texas, United States of America; Massey University, NEW ZEALAND

## Abstract

Macroscopic mechanical properties of human skin *in vivo* cannot be considered independent of adjacent subcutaneous white adipose tissue (sWAT). The layered system skin/sWAT appears as the hierarchical structural composite in which single layers behave as fiber-reinforced structures. Effective macroscopic mechanical properties of such composites are mainly determined either by the properties of the skin or by those of the sWAT, dependent on the conditions of mechanical loading. Mechanical interactions between the skin and the adjacent sWAT associated with a mismatch in the mechanical moduli of these two layers can lead to production of the skin wrinkles. Reinforcement of the composite skin/sWAT can take place in different ways. It can be provided through reorientation of collagen fibers under applied loading, through production of new bonds between existing collagen fibers and through induction of additional collagen structures. Effectiveness of this type of reinforcement is strongly dependent on the type of mechanical loading. Different physical interventions induce the reinforcement of at least one of these two layers, thus increasing the effective macroscopic stiffness of the total composite. At the same time, the standalone reinforcement of the skin appears to be less effective to achieve a delay or a reduction of the apparent signs of skin aging relative to the reinforcement of the sWAT.

## Introduction

Mechanical properties of human skin measured *in vitro* and *in vivo* can differ significantly [1], demonstrate significant inter-subject and inter-area variations *in vivo* [2], and are age-dependent [3,4]. Different procedures were developed to improve the mechanical properties of the skin and to reduce its altered appearance during aging. These procedures are mainly based on the reinforcement of the dermis through induction of excessive collagen structures [5]. At the same time, there is growing evidence that skin aging is not solely connected with a modification of the skin structure, but is also significantly dependent on the properties of the underlying subcutaneous white adipose tissue (sWAT) [5,6]. This affects not only the physiological, but also the mechanical properties of the skin/sWAT bilayer. Indeed, it is well appreciated that a stiff stressed thin layer interacting with a thick compliant substrate can produce different types of structural instabilities such as wrinkles, and this effect is strongly dependent on the mechanical properties of both layers [7].

The Young’s modulus describes the strain response of materials to uniaxial stress. Whereas some authors reported that the Young’s modulus of the skin *in vivo* is several orders of magnitude bigger than the corresponding modulus of the sWAT and thus should be almost independent of the mechanical properties of the subcutis [8], others stated that this modulus is generally dependent on the properties of sWAT [9]. The latter should be the case for at least some loading conditions, since the skin and sWAT layers are mutually confined *in vivo* [10], and their interface cannot slide past each other under mechanical deformation. Thus, the skin/sWAT system should be considered as a composite material containing at least two layers with distinct mechanical properties. Furthermore, the Poisson’s effect (expansion of material in the directions perpendicular to the direction of loading) in sWAT cannot be neglected. This Poisson’s effect was clearly described in experiments with biaxial loading where tissue elongations in two orthogonal directions mutually affected each other [11].

The skin/sWAT system should be considered as a hierarchical composite: the bilayer of skin and sWAT can be described as a structural layered composite, in which the single layers demonstrate the properties of fiber-reinforced structures [12]. Here, we analyze such hierarchical composite theoretically and describe its effective mechanical properties. We demonstrate that these properties are mainly determined either by skin or by sWAT features. Which one of the two prevails is mainly dependent on the conditions of mechanical loading applied to the skin surface. Reinforcement of only one layer cannot improve the stiffness of the composite for all types of mechanical loadings. These considerations are of particular importance in the context of anti-aging procedures. Such procedures should be aimed at reinforcing both the skin and sWAT.

## Effective mechanical characteristics of the multilayer composite of skin and sWAT

As shown in vacuum suction experiments, the stiffness of the reticular dermis is approximately three orders of magnitude bigger than the corresponding modulus of the upper skin [8,13]. SWAT is also not a single homogeneous layer, and the superficial and deep sWAT structures have different morphological and mechanical properties [6,14]. Moreover, Sommer and colleagues [11] reported that abdominal sWAT is stiffer in the areas proximal to the skin as well as in deeper regions (more than 30 mm from the skin) compared to its middle area, which means that sWAT should be biomechanically considered even as a three-layered structure. However, at a first approximation, we will further consider the composite skin/sWAT as a bi-layer system.

In a general case, the composite skin/sWAT will be further described as a multilayered system with different layers *i* having the thicknesses *d*_*i*_, Young’s moduli *E*_*i*_, and Poisson’s ratios *ν*_*i*_. This description does not take into account the anisotropic, viscoelastic and non-linear properties, and thus limits the further consideration to the case of low strains and low strain rates applied to such composite. Such multilayered structure can be reduced to a single layer having the thickness *d* = ∑_*i*_ *d*_*i*_ and some effective mechanical characteristics, *E*_*c*_ and *ν*_*c*_. However, these effective characteristics are very dependent on the type of loading as well as on the conditions between the boundaries of the layers.

### Composite skin/sWAT under longitudinal loading

**Fig 1:**
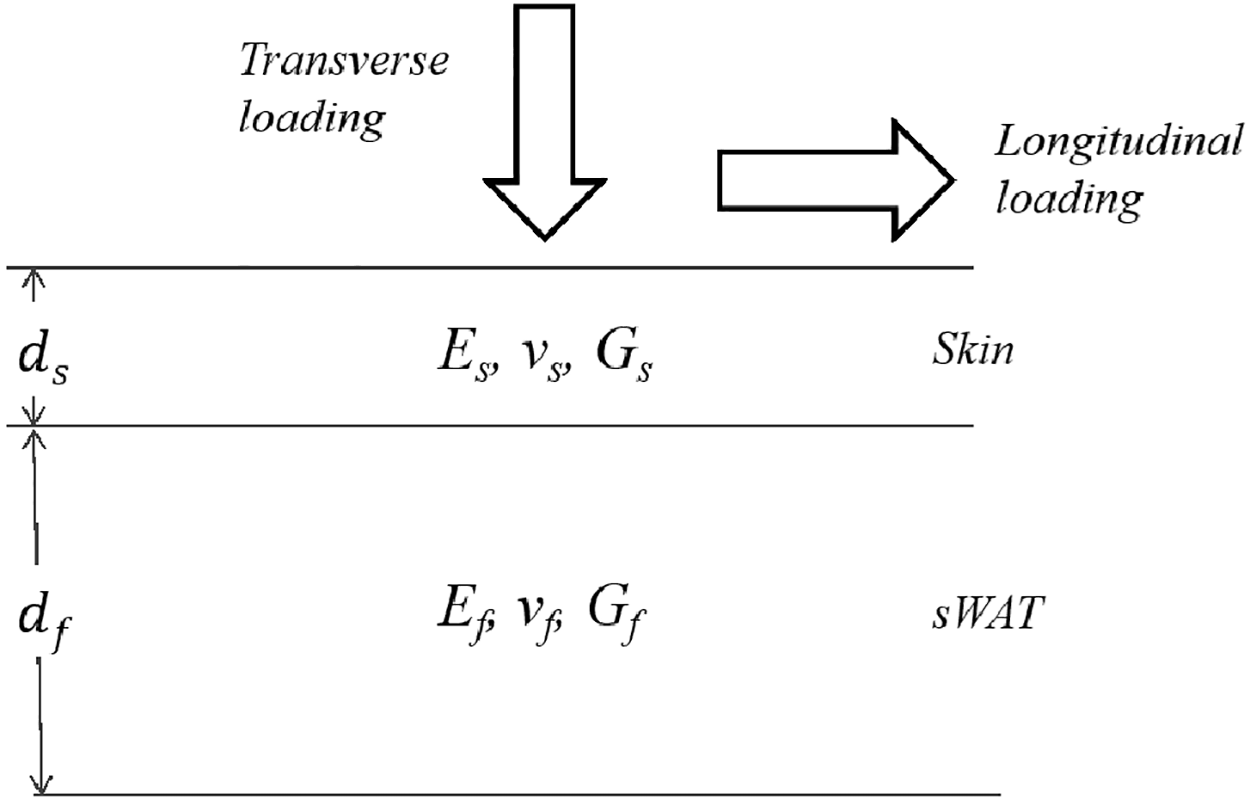
Structural composite skin/sWAT.

Let us consider the multilayer skin/sWAT composite which is subject to a longitudinal loading in the direction parallel to its surface (Fig 1). A simple solution for effective mechanical parameters of this composite can be found under iso-strain conditions, i.e. under assumption that all composite layers undergo the same deformation. After straightforward calculations, the effective Young’s modulus and Poisson’s ratio for such multilayer composite can be then presented in the form

$$E_{c}^{∥}=\frac{A}{d}(1-\nu_{c}^{2}),\;\nu_{c}=B/A$$ (1)

Here $A=\int_{0}^{d}\frac{E(z)}{1-\nu^{2}(z)}dz$, and $B=\int_{0}^{d}\frac{E(z)\nu (z)}{1-\nu^{2}(z)}dz$. Further, at a first approximation, we will consider the composite skin/sWAT as a bilayer system (Fig 1) containing a spatially homogenous skin (index *s*) and sWAT (index *f*) layers. Generalization to the case of a multilayer system is straightforward.

Assuming the Young’s moduli and the Poisson’s ratios in each layer are spatially constant, the effective Young’s modulus of such composites subjected to a longitudinal loading, $E_{c}^{∥}$, can be presented in a simple form

$$E_{c}^{∥}\approx \alpha E_{s}\frac{1-\nu_{c}^{2}}{1-\nu_{s}^{2}}+(1-\alpha )E_{f}\frac{1-\nu_{c}^{2}}{1-\nu_{f}^{2}}$$ (2)

where *α* = *d*_*s*_ / *d* is the relative thickness of the skin in the total composite skin/sWAT. This expression obviously reduces to the classical rule of mixtures in the case of *ν*_*s*_ = *ν*_*f*_.

$$E_{c}^{∥}\approx \alpha E_{s}+(1-\alpha )E_{f}$$ (3)

Whereas the iso-strain approximation provides simple results, this assumption is an oversimplification. Physically more realistic is the case of the mutual confinement between the layers, which means that the layers cannot slide past each other under mechanical deformation. In this case, strains in both layers at the interface of skin and sWAT should be the same, which provides the following effective Young’s modulus [15]:

$$E_{c}^{∥}=\alpha E_{s}+(1-\alpha )E_{f}+\frac{\alpha (1-\alpha )E_{s}E_{f}{\left(\nu_{s}-\nu_{f}\right)}^{2}}{\alpha E_{s}{\left(1-\nu_{f}\right)}^{2}+(1-\alpha )E_{f}{\left(1-\nu_{s}\right)}^{2}}$$ (4)

This expression reduces to the rule of mixtures Eq (3) at *ν*_*s*_ = *ν*_*f*_, i.e. when both composite’s layers demonstrate the same Poisson’s effect.

Typical thickness of the facial skin is about 1–1.5 mm [16], whereas the thickness of the adjacent sWAT layer can be several folds bigger. Thus, the typical value of *α* for the facial skin should be about 0.2–0.5. At low strain rates, the Young’s modulus of sWAT was estimated to be about 1 kPa; however, it can increase more than three orders of magnitude at higher strain rates [17,18]. Various values were reported for the Young’s modulus by different authors for the human skin at low strains and strain rates. The value of 78 kPa was found in [19], whereas the Young’s modulus in the cheek area of a fresh cadaver was reported to be 15 kPa [20]. *In vivo* measurements on the facial skin provided in [2] demonstrated significant inter-subject and inter-area variations with Young’s moduli ranging from approximately 40 kPa in the cheek area adjacent to the lips up to 65 kPa in the malar area. Thus, at low strain rates, *E*_*s*_ should be considered to be much bigger than *E*_*f*_. In this case, $E_{c}^{∥}\approx \alpha E_{s}$, and the effective Young’s modulus of the composite is mainly determined by mechanical properties of the skin and not by those of the sWAT. At the same time, at high strain rates, the stiffness of sWAT quickly increases and its contribution to the effective stiffness of a composite skin/sWAT can no longer be neglected [17].

### Composite skin/sWAT under transverse loading

Let us now consider the composite skin/sWAT subjected to a transverse loading (Fig 1). Its effective Young’s modulus can be simply found in the case of iso-stress loading with *ν*_*s*_ = *ν*_*f*_:

$$E_{c}^{⊥}=\frac{E_{s}E_{f}}{\alpha E_{f}+(1-\alpha )E_{s}}\approx \frac{E_{f}}{1-\alpha }$$ (5)

where we once more have taken into account that *E*_*f*_ ≪ *E*_*s*_. From Eq (5), the effective Young’s modulus $E_{c}^{⊥}$ of the composite skin/sWAT under iso-stress conditions is mainly determined by mechanical properties of its weaker sWAT layer.

Similar to the case of the iso-strain loading, the iso-stress condition is also not realistic. The effective Young’s modulus for the case of the mutual confinement of the layers can be presented in the form [15]:

$$E_{c}^{⊥}=\frac{E_{s}E_{f}}{\alpha E_{f}+(1-\alpha )E_{s}-\frac{2\alpha (1-\alpha ){\left(\nu_{s}E_{f}-\nu_{f}E_{s}\right)}^{2}}{(1-\nu_{s})(1-\alpha )E_{f}+(1-\nu_{f})\alpha E_{s}}}$$ (6)

For the case *E*_*f*_ ≪ *E*_*s*_ and *ν*_*f*_ < 0.5 (sWAT is compressible), expression Eq (6) can be reduced to a simple form, which evidently demonstrates the dependence of effective mechanical characteristics of such composites on the Poisson’s ratio for sWAT:

$$E_{c}^{⊥}\approx \frac{E_{f}}{1-\alpha }\frac{1-\nu_{f}}{1-\nu_{f}-2\nu_{f}^{2}}$$ (7)

The Poisson’s ratio for human fat tissue was reported to be about 0.4 [21]; skin, however, contains more water and collagen, and can be considered almost incompressible material with *ν*_*s*_ ≈ 0.47 − 0.49 [19,22,23]. From Eq (7), we can receive an estimation for the effective Young’s modulus for transversal loading of the composite skin/sWAT with *ν*_*f*_ = 0.40 in the case *E*_*f*_ ≪ *E*_*s*_:

$$E_{c}^{⊥}\approx 2.14\frac{E_{f}}{1-\alpha }$$ (8)

This modulus rapidly increases with the Poisson’s ratio of sWAT, being 1.60*E*_*f*_ / (1 − *α*), 3.77*E*_*f*_ / (1 − *α*), and 8.78*E*_*f*_ / (1 − *α*) for *ν*_*f*_ = 0.35; 0.45; and 0.48, respectively.

From Eq (6), it can be easily shown that at *ν*_*f*_ → 0.5 (sWAT is almost incompressible), *ν*_*s*_ ≈ 0.5, and *E*_*f*_ ≪ *E*_*s*_, the effective Young’s modulus $E_{c}^{⊥}$ of the composite skin/sWAT will be mainly determined by mechanical characteristics of the skin:

$$\underset{\nu_{s},\nu_{f}\to 0.5}{\text{lim}}E_{c}^{⊥}=\alpha E_{s}$$ (9)

This can be the case for the sWAT containing a significant amount of water and/or of collagen. One important example could be the application of hyaluronan-based fillers which are normally injected subcutaneously, attract water and make the sWAT locally almost incompressible. Thus, injection of hyaluronan-based fillers not only produces the classical volume restitution, but can also—due to the Poisson’s effect—make the local mechanical properties of the composite skin/sWAT dependent on the skin and not on the sWAT characteristics. This should increase the effective stiffness of this composite making it more resistive to structural modifications under mechanical loading.

Thus, under physiological conditions, the Young’s modulus of the skin/sWAT composite is mainly determined by mechanical properties of the skin layer under mechanical loading parallel to the skin surface, and by sWAT properties under mechanical loading perpendicular to the skin. Such composites should generally be stiffer under transverse compression Eq (6) than under longitudinal loading Eq (4). At the same time, reinforcement of sWAT with non-compressible collagen structures and/or water can lead to substantial involvement of the skin in mechanical properties of the skin/sWAT composite under transverse mechanical loading.

### The role of sWAT in skin wrinkling

Subcutaneous adipose tissue is involved in the skin aging processes to a very substantive degree. Interactions between the sWAT and the skin is realized through an interplay between superficial adipocytes and fibroblasts from the reticular dermis, as well as through transformation of adipocytes to the synthetically highly active myofibroblasts, which can significantly influence the local synthetic activity for collagens and thus effectively modulate the stiffness of the skin layer [6]. At the same time, there is a direct mechanical interaction between the skin and the sWAT, which can influence the appearance of signs of skin aging. This mechanical interaction is connected with a mismatch in the Young’s moduli of the skin and sWAT at the interface skin/sWAT, which can lead to the induction of instabilities in the stiffer skin layer followed by production of cracks and wrinkles in the skin.

From the general theory of cracking of layered materials, we appreciate that under stress, the mechanical stability of the stiff thin film located on the surface of the thick compliant substrate is dependent on the stress value, the Young’s and Poisson’s moduli of both layers, as well as on the film thickness [7]. Stress in the skin layer can have different origins and can be of intrinsic, thermal or mechanical nature. The skin/sWAT composite, in which the skin presents the stiff film and the sWAT is the soft substrate, complies with such a model of layered materials. In accordance with this model, there should be a critical skin thickness $h_{s}^{cr}$ that allows for the production of full skin thickness wrinkles, which can be described as [24]

$$h_{s}>h_{s}^{cr}=\frac{2{\bar{E}}_{s}\Gamma_{s}}{\pi \sigma^{2}(1-\nu_{s}^{2})g(\xi )},\;g(\xi )\approx \frac{1.258-0.4\xi -0.26\xi^{3}-0.3\xi^{4}}{1-\alpha }$$ (10)

where *σ* is the stress in the skin; Γ_*s*_ is the toughness of the skin;$\xi =({\bar{E}}_{s}-{\bar{E}}_{f})/({\bar{E}}_{s}+{\bar{E}}_{f})$, and ${\bar{E}}_{i}=E_{i}/(1-\nu_{i}^{2})$. The parameter *ξ* measures the mismatch in the plane tensile modulus across the interface skin/sWAT and can theoretically vary in the range [-1;1] between the very “soft” and very “stiff” skin compared to sWAT. It should be noted that the human skin normally does not demonstrate a wrinkling across the full thickness [25], such that the relations Eq (10) cannot be applied directly and should be modified for the case of a partially cracked film [26]. Whereas such modifications would change the absolute values of the critical skin thickness, the qualitative results are the same.

From Eq (10), the “mechanical” production of wrinkles can take place only in skin in which the thickness exceeds some critical value. This threshold value increases with reducing Young’s modulus of the skin, with increasing stress applied to the skin and with increasing mismatch in the Young’s moduli of the skin and sWAT. Accordingly, the probability of the skin wrinkling can be reduced by reinforcement of the skin and/or sWAT, whereas the end result should be dependent on the absolute value of *E*_*s*_ / *g*(*ξ*). Indeed, the function *g*(*ξ*) quickly and non-linearly increases in the range *ξ* ∈ [0.5;1.0] [26]. This corresponds to the physiological values for the skin/sWAT composite. Thus, the standalone reinforcement of the mechanical properties of the skin through increasing Young’s modulus *E*_*s*_ should be not so effective as it looks at the first glance. From Eq (10), the increase of the skin’s Young’s modulus from 50 kPa up to 100 kPa (100%) will effectively increase the threshold of the skin thickness to wrinkling only by 10.3%. At the same time, the standalone mechanical reinforcement of sWAT will generally decrease the value of *g*(*ξ*) and effectively increase the threshold level of the skin thickness for wrinkling. For example, increase of *E*_*f*_ from 1 kPa up to 2 kPa by the fixed value of *E*_*s*_ = 50 kPa will increase the threshold of the skin thickness for wrinkling by approximately 70%.

Thus, the mechanical properties of the sWAT are substantially involved at least in mechanical aspects of the skin aging. At the same time, simple mechanical reinforcement of the dermis without concurrent reinforcement of sWAT will not necessarily lead to a significant delay or reduction of the signs of aging.

To understand how the skin and sWAT can be reinforced, we should analyze the mechanical moduli of these two layers from a biomechanical viewpoint.

### The dermis as a fiber-reinforced composite

So far, we have considered both the skin and the sWAT as spatially homogeneous layers. We now take into account that the dermis contains a three-dimensional collagen network with mechanical properties that significantly differ from the corresponding properties of its matrix. Application of physical forces to the skin can reinforce this network inducing production of additional fibrotic structures whose orientation should be dependent on the direction of applied mechanical loading.

Whereas the reinforcement of the dermis with fibrillar collagen bundles can improve its mechanical characteristics, the absolute value of this reinforcement should be strongly dependent on the orientation and volume fraction occupied by these reinforcing structures. Considering the reinforcing collagen bundles to be non-deformable in transverse direction, the effective Young’s modulus in the direction parallel to the bundles can be presented in the form of the rule of mixtures:

$$E_{s}^{∥}=(1-\beta )E_{sm}+\beta E_{col}$$ (11)

where *E*_*sm*_ is the Young’s modulus of the skin matrix without fibrotic structures, *E*_*col*_ is the Young’s modulus of the collagen bundles in the dermis which can be as high as 1 GPa [17], and *β* is the volume fraction occupied by dermal collagen.

Similar to Eq (5), the effective Young’s modulus measured perpendicular to the collagen bundles can for be presented in the form (*E*_*sm*_ ≪ *E*_*col*_)

$$E_{s}^{⊥}=\frac{E_{sm}E_{col}}{\beta E_{sm}+(1-\beta )E_{col}}∼\frac{E_{sm}}{1-\beta }$$ (12)

Thus, collagen fibers in the dermis oriented parallel to the skin surface cannot significantly reinforce the skin against mechanical loading applied perpendicular to the skin. Maximum reinforcement of the dermis for such loading can be achieved for fibers oriented perpendicular to the interface skin/sWAT. Such behavior was reported for elastin-like protein matrix reinforced with collagen microfibers, where the Young’s modulus measured in the direction of collagen orientation was six-fold bigger than the corresponding modulus measured in transverse direction [27].

Spatially homogenous 3D collagen networks in the skin should provide a reduced reinforcement comparing with an optimal case of the fibers being perfectly aligned in the direction of loading. In the case of spatially random and uniformly oriented single fibers, this corresponds to just 20% of its maximum value [28]. At the same time, collagen fibers in the dermis can quickly be reoriented in the direction of applied uniaxial load [29]. Such a collagen network adaptation to the loading can reinforce the skin several times and should serve as a first line response of the dermis to the mechanical loading.

Another mechanism for reinforcement of the collagen network is the recently described self-reinforcement of fiber networks under cyclic compressive loading through production of new bonds between the existing fibers [30]. This reinforcement mechanism is more pronounced under compressive than under tensile or shear loading and appears to be very effective: increases in bond density by about 3% lead to approximately a 10-fold increase in material stiffness.

The third reinforcement mechanism deals with an increase of the collagen density through production of new cross-linked collagen fibers. Considering the skin as a fiber-reinforced composite with fibrous bundles having an orientation between the spatially uniform 3D structure and full alignment in the direction of loading, the effective Young’s modulus of the skin can be estimated as

$$(1-\beta )E_{sm}+0.2\gamma \beta E_{col}\leq E_{s}^{∥}\leq (1-\beta )E_{sm}+\gamma \beta E_{col}$$ (13)

Here, we have taken into account the length correction factor *γ* < 1. According to [31], this factor is strongly dependent on the length of the single fibers, their diameter, inter-fibers spacing and interface bond strength between the fibers and the matrix, and can be theoretically described by the following relations:

$$\gamma =1-\frac{\text{tanh}\left(a\bar{L}/D\right)}{\left(a\bar{L}/D\right)},\;a=\sqrt{\frac{E_{sm}}{(1+\nu_{sm})E_{col}\text{ln}\left(\Lambda /D\right)}}$$ (14)

Here, $\bar{L}$ is the average length of the reinforcing fibres which can reach several mm; *D* is the diameter of the fibers which in porcine and human skin should be about 0.06–0.1 μm [32]; Λ is the average spacing between the fibers; *ν*_*sm*_ is the Poisson’s ratio for the skin matrix. From Eq (14), the length correction factor quickly diminishes with reduction of the length of collagen fibers, which leads to a weaker skin reinforcement. Whereas *E*_*sm*_ ≪ *E*_*col*_, and thus *a* ≪ 1, the ratio $\bar{L}/D$ can be bigger than 10^4^, which means that the length correction factor *γ* is not evidently ≪ 1. In other words, the second term in the expression Eq (13) can indeed improve the mechanical properties of the dermis; however, this can be the case only if the induced reinforcing structures have a high length correction factor. Induction of an additional network of mature collagen in the dermis should be considered as the second type of reinforcement reaction of the dermis following the quick reorientation of existing collagen fibers under loading.

### Mechanical properties of sWAT

There are two different types of collagen networks in sWAT, which are known as inter- and pericellular fibrotic structures [12]. Intercellular networks contain the fibrillar collagens of the types I and III, whereas the pericellular network mainly consists of the collagens of the types IV and VI. It was theoretically predicted that the mechanical properties of sWAT are mainly determined by pericellular and not by intercellular fibrotic structures [17]. The lack of collagen VI reduces the tensile strength of sWAT in the knockout mice up to 50% [33]. At the same time, investigation of the sWAT with multiphoton microscopy revealed its very heterogeneous response to tensile strain on a microscopic scale [34]: whereas in some areas significant loads were imposed on the intercellular fibers, in other areas the cells were distorted and their pericellular structures deformed.

Adipocyte size as well as fibrotic structures around the cells demonstrate big variations, mainly related to the morphological type of sWAT [35]. These variations were clearly observed with scanning electron microscopy in different facial sWAT compartments [36]. It was reported that the labial and nasal sWAT compartments typically contain mature adipocytes embedded in a dense collagen matrix; such morphological structures were classified as “fibrous” fat [37]. At the same time, the malar and periorbital fat compartments demonstrate a “structural” morphology with lobules of mature adipocytes which are homogenously covered by thin collagen fibres producing the basket-like structures. On the contrary, morphological structures of the deep buccal fat compartment are characterized by large single adipocytes that are not completely covered with pericellular collagen, which is typical for the fat of a “store” type.

Intercellular collagen fibers in sWAT produce a coarse-mesh structure with a typical unit size of several millimeters which has no closed boundaries and thus can be considered as an open-cell foam. This network appears as a homogenous septum without any preferred orientation [11]. Under normal conditions, the volume fraction of intercellular fibrosis in sWAT should be very small. However, this fraction can be much higher in compartments known as “fibrous” fat compartments [35]. Since the inter-fiber distance for intercellular fibrotic structures in sWAT is much bigger than the corresponding value in the dermis, the length correction factor for these structures in sWAT should be *γ* ≪ 1, which significantly reduces the contributions of intercellular fibrosis into the total mechanical properties of sWAT.

The main reinforcement of sWAT is connected with its pericellular fibrotic structures appearing around the single adipocytes. Microstructure in which the harder phase (pericellular fibrosis) encapsulates the softer phase (adipocytes) can be described by the upper bound of the Hashin-Shtrikman model [38]:

$$E_{{}_{f}}^{HS}=E_{pf}\frac{\mu E_{pf}+(2-\mu )E_{tg}}{\mu E_{tg}+(2-\mu )E_{pf}}$$ (15)

where *E*_*pf*_ is the Young’s modulus of the reinforced basement membrane, which was assessed to be about 10 kPa [17]; *E*_*tg*_ is the Young’s modulus of the triglycerides in adipocytes which is less than 0.1 Pa; *μ* is the volume fraction of sWAT occupied by pericellular fibrosis. Since *E*_*tg*_ ≪ *E*_*pf*_, expression Eq (15) can be reduced to $E_{f}^{HS}=\mu E_{pf}/(2-\mu )$. It is seen that $E_{f}^{HS}$ monotonously increases with *μ*.

For the cells of the radius *R* surrounded by pericellular fibrosis of the thickness *h*, the volume fraction *μ* can be estimated as *μ* ≈ 3*h* / *R* [12], which provides the Hashin-Shtrinkman’s upper limit for the Young’s modulus of

$$E_{f}^{HS}=3hE_{pf}/2R$$ (16)

Here *R* should be considered as the average value $\bar{R}$ obtained for the whole adipocytes’ population in sWAT [12]. Similar estimation for the Young’s modulus of sWAT in the form $E_{f}\approx \frac{h}{\bar{R}}E_{pf}$ was obtained for the model of a closed-cell foam in [12,17].

From Eq (16), the effective Young’s modulus of sWAT mainly containing pericellular fibrosis linearly increases with the thickness of the reinforced basement membrane and decreases with increasing average adipocyte radius. Typical values of *h* can vary between 0.2 μm and 2 μm [17]; the typical average radius of an adipocyte in different facial compartments is about 25–50 μm. Thus, the ratio $h/\bar{R}$ can vary in the range of 0.004–0.080. From here, thickening of the reinforced basement membrane and/or reduction of adipocyte sizes in the physiological range can indeed reinforce the sWAT.

### Adaptation of sWAT structure to mechanical loading

Mutual confinement of the layers exists not only between the skin and sWAT, but also between the sWAT and deeper tissue layers. In most body areas, this layer is composed of muscles, in which the Young’s modulus is strongly dependent on their state (i.e. tension or relaxation) and varies for different measurement procedures between 1 and 100 kPa. For example, the Young’s modulus of relaxed *rectus femoris* was reported to be in the range of 10–30 kPa [39]. Since the mechanical characteristics of this third layer are similar to that of the skin, the influence of this layer on the effective mechanical characteristics of the total composite should be mainly connected with an increase of the relative skin thickness, *α*.

A different situation can be observed in the scalp area where the layer adjacent to sWAT is the *galea aponeurotica*, which has a tendon-like structure with a Young’s modulus of about 1 GPa typical for a tendon [40]. Deformation of such a three-layer (skin/sWAT/galea) composite structure of the scalp having a full mutual confinement between the sWAT and *galea aponeurotica* can lead to a significant deformation of the sWAT layer which can exceed its ultimate tensile strength and thus lead to a mechanical break of this tissue. To resist this deformation, sWAT layer in the scalp area should be mechanically strongly reinforced. Since the effective Young’s modulus of an elastic layer (sWAT) confined between two rigid layers (skin and galea) can be described by expression Eq (7), such reinforcement can take place through increase of *E*_*f*_ and/or of the Poisson’s ratio *ν*_*f*_. Indeed, sWAT in the scalp area was found to be a rigid structure containing multiple compartments separated by septa [41]. Very recently, we have proposed that such mechanical reinforcement of sWAT together with androgen-dependent transition of adipocytes from this layer to myofibroblasts can be an important pathophysiological step in pattern baldness typical for androgenetic alopecia [42].

This may be the consequence of a mutual confinement of different tissue layers and should be considered a slow adaptation of the sWAT structure to mechanical loading, in contrast to a quick adaptation produced by reorientation of available collagen fibers in the direction of this loading. Indeed, fibroblasts normally exist in an environment of low stiffness, whereas the existence of highly synthetically active myofibroblasts is characteristic for medium and high substrate stiffness; moreover, mechanical stress is able to induce the differentiation of fibroblasts into myofibroblasts [43]. Similarly, transforming growth factor β was reported to be upregulated by mechanical stress in different cell types, including adipocytes. Such an upregulation is connected with the production of additional collagens and other extracellular matrix proteins in adipose tissue [44]. Thus, adaptation of the sWAT stiffness to mechanical stress can be realized through well-known physiological pathways.

## Discussion

The hierarchical skin/sWAT composite should demonstrate different mechanical properties under various conditions of mechanical loading. According to Eq (3), for mechanical loading oriented parallel to the skin surface, the resistance of such a composite structure should be determined mainly by cutaneous mechanical characteristics. This could be, for example, the case for the loading connected with gravity forces.

Whereas the mechanical properties of the composite skin/sWAT can be generally improved through some modification of fibrotic structures in the dermis, the degree of this improvement is dependent on different factors. First, reinforcement of the skin can be achieved only by a significant increase of the volume fraction *β* occupied by reinforcing fibers in the dermis. Second, from Eq (14), the absolute value of this reinforcement is dependent on the length correction factor *γ*, which can be increased through induction of new bonds between the fibers [30]. This means that anti-aging treatment procedures should be differentiated according not only to the amount of induced fibrotic structures, but also to the reinforcing quality of these structures.

It should be taken into account that mechanical modification of the collagen network is strongly dependent on its volume behavior during deformation: under volume-preserving conditions (which are typical for the tissues containing tightly packed cells), such networks resist the mechanical loading differently than in the case of a network that can easily change its volume [45,46]. Recently it was demonstrated that the Young’s modulus of a collagen network can be an order of magnitude lower under compression than under extension [46]. Moreover, the stiffness of such a network could be strongly modified if the uniaxial strain was combined with a shear deformation. At the same time, it was reported that compression is much more effective for the induction of new bonds between the fibers than tensile or shear deformation [30]. This clearly demonstrates that different types of mechanical loadings can provide not only quantitatively but also qualitatively different reinforcing effects.

For mechanical loading directed perpendicular to the skin surface, macroscopic mechanical behavior of the hierarchical composite skin/sWAT with mutually confined layers should be mainly dependent on the mechanical properties of the sWAT layer. From Eqs (8) and (16),

$$E_{c}^{⊥}∼\frac{3h}{2(1-\alpha )\bar{R}}\frac{1-\nu_{f}}{1-\nu_{f}-2\nu_{f}^{2}}E_{pf}$$ (17)

From Eq (17), resistance of the composite skin/sWAT to mechanical loading oriented perpendicular to the skin surface increases with the thickness of the pericellular fibrosis around single adipocytes and with progressive reduction of adipocytes’ sizes. It is also inversely dependent on the relative thickness of sWAT in the total composite structure. This resistance is also very sensitive to the Poisson’s ratio in sWAT: increase of *ν*_*f*_ from 0.4 up to 0.45 or 0.48 will change the Young’s modulus of the composite 1.76-fold or 4.10-fold, respectively. Taking into account the ability of sWAT to reinforce its structure through induction of additional fibrotic structures under mechanical loading, such modification of the sWAT structure should be an important target in anti-aging procedures.

As an example, let us consider the treatment procedures based on application of the vacuum massage. On the one hand, application of the vacuum to the skin does not influence the skin thickness. However, it significantly increases the thickness of sWAT [47]. This should lead to a reduction of the relative thickness of the skin *α* in the total skin/sWAT composite, and thus should lead to a reduction of its effective Young’s modulus. On the other hand, significant increase of the fractional volume occupied by intercellular fibrotic structures in abdominal sWAT was observed after long-term application of the vacuum [48], which points to the adaptive character of this reaction. The end result of this and similar treatment procedures should be dependent on the ratio between these two reciprocal processes, and thus can vary in different subjects.

Standalone reinforcement of the skin should be in a general case by far not so effective in anti-aging procedures as the corresponding reinforcement of the sWAT. Whereas the doubling of the Young’s modulus of the skin effectively increase the thickness threshold of the skin to wrinkling only by about 10%, doubling of the Young’s modulus of the sWAT can cause 1.7-fold increase of this parameter, thus making the composite skin/sWAT much more stable to appearance of these aging signs. This can clearly shift the point of interest in anti-aging from the dermis reinforcement to the sWAT modification.

Because of the big dispersion of the facial skin thicknesses [16] as well as of the high variability of the adjacent sWAT structures in different facial sWAT compartments [35], skin aging processes leading to the optical modification of the skin and wrinkles production should be markedly distinct in various facial areas. Facial sWAT compartments are age-dependent and demonstrate a significant reduction of their fibrotic content with progressing aging [35]. At the same time, the deep facial buccal fat compartment contains only a very limited amount of pericellular fibrosis [36], and it can be assumed that such structures cannot be significantly induced in this fat compartment under quasi-physiological conditions. On the contrary, the labial fat compartment normally contains a significant amount of pericellular fibrosis, and it can be supposed that additional amount of reinforcing structures can be induced in this compartment by proper stimulation. These features can at least partly explain the spatial differences in facial skin rigidness observed in [2]. Consequently, the anti-aging strategy should take into account the morphological and physiological peculiarities of the sWAT compartments which belong to the corresponding local skin/sWAT composites and thus should be generally different in various facial areas.

This conclusion should be correct not only for the anti-aging procedures based on the mechanical stimulation of the composite skin/sWAT. Since the main pathways for the radio-frequency (RF) currents in sWAT are connected with fibrotic structures in this tissue [16,49], they should be selectively heated during RF procedures. Such local heating can induce additional production of fibrotic structures in sWAT which will increase the mechanical resistance of the total composite skin/sWAT.

Recently, it was recognized that the superficial layer of sWAT adjacent to the dermis contains a special pool of adipocytes named dermal adipocytes which are characterized by high turnover rates. These cells are involved in such different processes as hair growth, wound healing, thermoregulation, innate immune reaction, and some skin efflorescences. [6,14,50,51]. This layer was very recently reported to be especially sensitive to mechanical stress and be able to induce the adaptation of its extracellular matrix to mechanical loading [52,53]. This makes the superficial layer of sWAT an important target in anti-aging procedures [5]. In this context, especially interesting is the ability of dermal adipocytes to undergo the transformation into the myofibroblasts [54], and, as it was very recently proposed [14] and demonstrated [55], that this transformation should be reversible. It was also proposed that the process of adipocyte-myofibroblast transformation is involved in skin aging [5], and that this process is mechanosensitive [42]. Since this superficial sWAT area represents the transition zone characterized by a mismatch in mechanical moduli of the two adjacent layers, the cells from this area will be exposed to the strong gradients of mechanical stresses, which are dependent on the local mechanical properties of the skin and sWAT near the interface between these layers. This puts a new complexion on the connection between the skin and sWAT in aging.

One limitation of this model is that we considered the composite skin/sWAT as a bi-layer. This approach does not take into account different mechanical characteristics of such skin layers as stratum corneum, epidermis, papillar and reticular dermis, which are known to be different, and consider the skin as a homogeneous isotropic layer with some effective mechanical properties. Generalization of the model considered in this article to the case of the multi-layer skin will be provided elsewhere.

## Conclusion

The skin/sWAT system *in vivo* demonstrate mechanical properties that are strongly dependent on the conditions of its mechanical loading. Biomechanically, skin and sWAT *in vivo* should be considered as the single hierarchical composite structure containing layers, reinforced by collagen structures. For mechanical deformations provided in direction parallel or perpendicular to the skin surface, macroscopic mechanical stiffness of this composite is mainly determined by the mechanical properties of the skin or by those of the sWAT, respectively. Since the skin is normally subjected to mechanical forces of different orientations, generally, the mechanical response of such a composite stress should be in between these two values, providing the lower and upper limits for its possible mechanical response. From this viewpoint, the standalone reinforcement of the skin properties -the cornerstone of the majority of anti-aging procedures—is not optimal and should be combined with a correspondent mechanical reinforcement of the sWAT structure.

The critical thickness of the skin, which, under stress, can provide the loss of its mechanical stability and allows the production of dermal wrinkles, is dependent on the stiffness of both composite layers and can be significantly increased by even relatively small reinforcement of the sWAT. This can build the theoretical background for preventive anti-aging treatments. At the same time, the relative reinforcement of single layers in the composite skin/sWAT is different in various anti-aging procedures, which can significantly influence the treatment results.

The reinforcement of these two structurally different layers takes place in different ways. While the single application of mechanical stress to the skin causes the reorientation of collagen fibers under loading, thus producing its quick albeit limited reinforcement, repeated mechanical loadings can cause the adaptive reinforcement of the dermis through induction of new bonds between the collagen fibers, or even of additional intercellular collagen networks. At the same time, the simple increase of the volume fraction occupied by dermal collagen is not enough for significant mechanical reinforcement of the dermis. For this, the collagen bundles should have some critical length and diameter as well as a significant interface bond strength between the fibres and the matrix.

On the contrary, reinforcement of the sWAT under mechanical loading can be connected with induction of additional pericellular fibrotic structures around single adipocytes and/or adipocyte clusters, as well as with effective reduction of the average adipocyte size. Reinforcement of sWAT appears to be much more effective in anti-aging procedures than the standalone reinforcement of the skin. This should be evidently taken into account for the formulation of anti-aging strategies.
